# Temperature-Dependent Microstructural Evolution of Seed-Assisted Low-Template ZSM-5 and Its Catalytic Behavior in Benzene Alkylation with Methanol

**DOI:** 10.3390/molecules31173084

**Published:** 2026-09-02

**Authors:** Shuyi Wu, Yanji Shi, Qihao Zheng, Jianle Yuan, Yilin Xiang, Yining Jiang, Jiaxing Zhang, Ajuan Zhou, Xinwen Guo

**Affiliations:** 1 School of Chemical Engineering, Ocean and Life Sciences, Dalian University of Technology, Panjin 124221, China; wsy021812@mail.dlut.edu.cn (S.W.);; 2 State Key Laboratory of Fine Chemicals, Frontiers Science Center for Smart Materials, School of Chemical Engineering, Dalian University of Technology, Dalian 116024, China; 3 Liaoning Key Laboratory of Chemical Additive Synthesis and Separation, Yingkou Institute of Technology, Yingkou 115014, China

**Keywords:** ZSM-5 zeolite, seed-assisted low-template synthesis, growth mechanism, alkylation, structure–activity relationship

## Abstract

Low-cost synthesis of ZSM-5 zeolite is critical for its large-scale industrial application. Herein, a seed-assisted low-template hydrothermal route was employed to fabricate ZSM-5 zeolites. The regulatory effects of gradient crystallization temperatures on the crystallization kinetics, morphology, pore structure and acid properties of ZSM-5 were systematically investigated, and the structure–activity relationship linking temperature-driven evolution and catalytic behavior in benzene alkylation with methanol was established. The results reveal that increasing the crystallization temperature shortens the amorphous induction period, facilitates framework aluminum (Al) incorporation and increases the concentration of Brønsted acid sites. Meanwhile, crystallization temperature modulates the specific surface area and intercrystalline mesopore volume, thereby tuning the accessibility of acid sites and the mass transfer efficiency of reactants and products. The sample synthesized at a moderate temperature of 170 °C achieves an optimal balance between textural properties and acid concentration. Benefiting from its large specific surface area, abundant intercrystalline mesopores and high Brønsted acid concentration, the catalyst affords a benzene conversion of 69.1% and a methanol utilization of 79.1%, and maintains stable operation over 135 h on stream. The temperature-dependent regulation insights uncovered in this work facilitate low-cost synthesis of ZSM-5 and rational design of high-performance catalysts for aromatic alkylation.

## 1. Introduction

Light aromatic hydrocarbons including benzene, toluene and xylene (BTX) are indispensable petrochemical feedstocks for manufacturing high-value chemicals, fine intermediates and high-performance polymers [1,2]. Global demand for xylene sustains steady growth. Conventional aromatic production heavily relies on petroleum-based catalytic reforming and thermal cracking, which is restricted by limited crude oil resources [3,4,5]. Benzene alkylation with coal- or biomass-derived methanol serves as an economical and strategic pathway to alleviate the supply–demand imbalance of aromatics [6,7]. ZSM-5 zeolite (MFI-type) features three-dimensional intersecting ten-membered ring channels and tunable acidity, exhibiting superior shape-selective catalysis and broad substrate adaptability, thus becoming the dominant catalyst for benzene–methanol alkylation [8,9,10,11,12,13].

Conventional ZSM-5 synthesis heavily relies on organic structure-directing agents (OSDAs), which guide zeolite crystallization via electrostatic interactions and space-filling effects. Nevertheless, this template-mediated route suffers from high OSDAs cost, nitrogen-containing wastewater discharge, and mandatory high-temperature calcination for OSDAs removal—a process that consumes substantial energy and releases CO_2_ and NO_x_ emissions. These drawbacks raise both economic and environmental concerns, motivating the development of more sustainable synthesis strategies [14,15,16,17]. Driven by green chemistry principles and industrial economic demands, seed-assisted low-template or template-free synthesis has emerged as a promising research direction for low-cost ZSM-5 preparation [18]. However, the lack of template-derived space-filling and induction effects readily generates the co-crystallized MFI/MOR zeolite and thermodynamically dense impurity phases such as quartz, which pose huge challenges to the precise crystallization control of ZSM-5 zeolites [15].

To address the above crystallization challenges in low-template systems, crystallization temperature has been proven to be a pivotal thermodynamic parameter that modulates the physicochemical properties and catalytic behavior of zeolites [19,20,21]. It governs the depolymerization of aluminosilicate oligomers and assembly kinetics of nanocrystallites, thereby tuning crystal morphology and pore networks. Moreover, crystallization temperature directly regulates the extent of framework Al isomorphic substitution, which further adjusts the concentration and spatial distribution of acid sites [22]. Despite these known effects, the detailed mechanisms by which temperature modulates crystallization kinetics, framework Al incorporation, mesopore evolution and acid properties remain poorly understood specifically for seed-assisted low-template systems. More importantly, the structure–activity relationship linking temperature-driven evolution to catalytic performance in benzene–methanol alkylation has not yet been systematically clarified.

Herein, the hydrothermal crystallization temperature was set as a single thermodynamic variable to systematically investigate its regulation mechanisms on the low-cost synthesis, morphology, framework structure and acid properties of ZSM-5 zeolite. By correlating zeolite structural evolution with catalytic performance in benzene–methanol alkylation, this study aims to reveal the precise balance mechanism among crystallinity, morphology, pore structure and acid concentration.

## 2. Results and Discussion

### 2.1. Crystallization Mechanism of ZSM-5 Synthesized at Different Temperatures

To systematically investigate and elucidate the growth mechanism and structural evolution of ZSM-5 zeolite under varying crystallization temperatures, this work expanded the temperature gradient to 90 °C, 120 °C, 170 °C and 200 °C, and performed time-resolved sampling to evaluate the control rules of temperature on MFI phase formation.

Figure S1 illustrates that at a low temperature of 90 °C, the product remains fully amorphous without any detectable MFI diffraction signals, even after static aging for 144 h. This thermodynamically verifies that the thermal energy provided at 90 °C fails to reach the activation energy threshold required to initiate the crystallization of this seed-assisted low-template aluminosilicate gel system.

**Figure 1:**
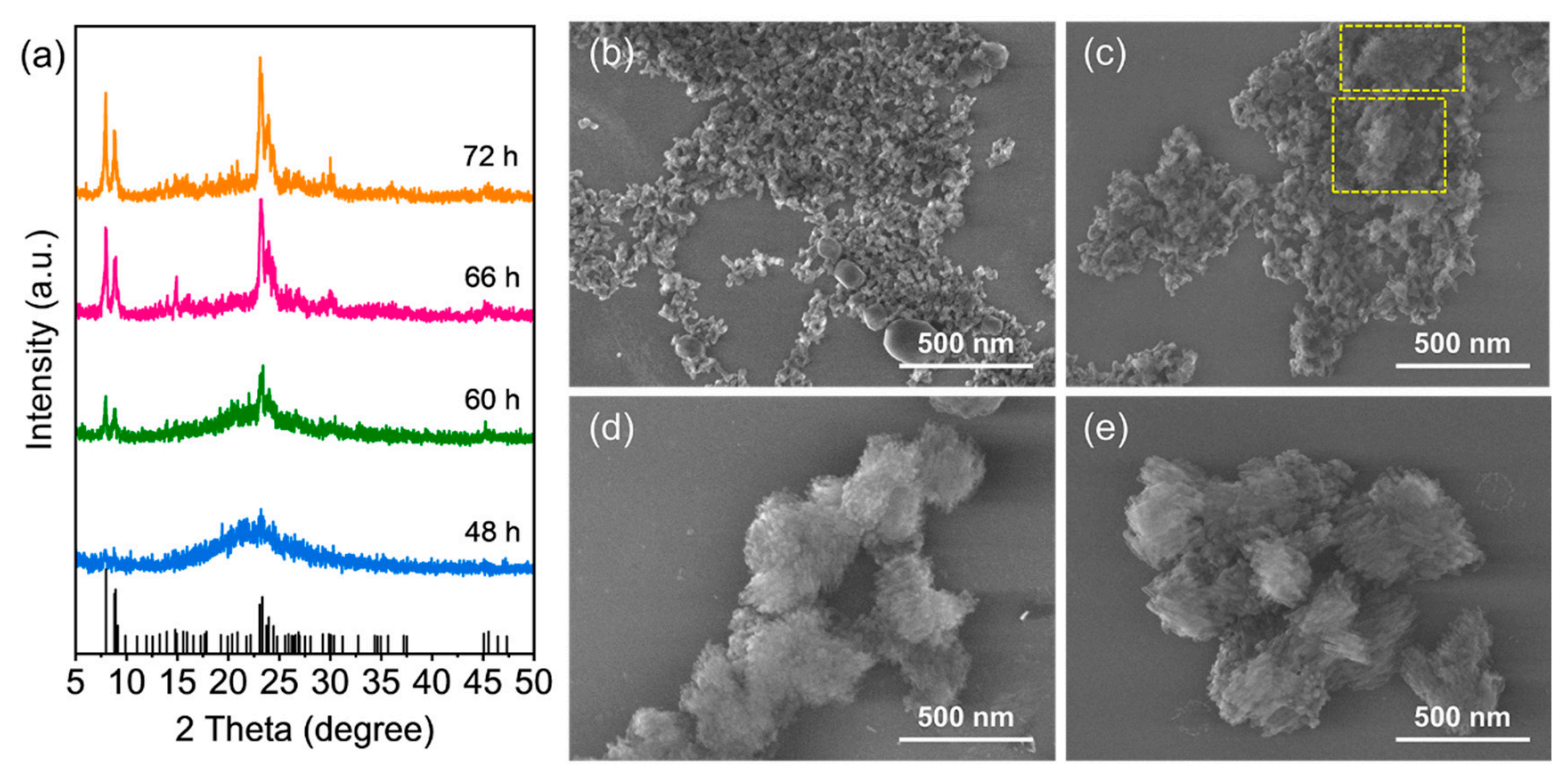
Characterization of ZSM-5 (120 °C) collected at various crystallization times. (**a**) XRD patterns. SEM images: (**b**) 48 h, (**c**) 60 h, (**d**) 66 h, and (**e**) 72 h.

Figure 1 shows the powder XRD patterns and SEM images of as-synthesized ZSM-5 (120 °C) samples collected at different crystallization times. As seen, in the early and prolonged intermediate stages (0–48 h), only a broad amorphous peak can be observed in all XRD patterns. At the relatively low thermodynamic driving force of 120 °C, the dissolution, local rearrangement, and depolymerization–polycondensation of the precursor gel at the liquid–solid interface exhibit slow kinetic characteristics. Figure 1b also indicates a prevalence of amorphous substances and sparse small crystallites. By comparing the morphology and particle size, these crystals are identified as the pre-introduced S-1 seed crystals. As the crystallization time progresses to 60 h, characteristic MFI diffraction peaks at 7.9°, 8.8°, 23.1°, 23.9°, and 24.5° gradually appear, marking the onset of crystal growth [23]. Figure 1c captures the partial dissolution of seed crystals and the incipient formation of nanoaggregates (marked with a yellow dotted line box), accompanied by the continuous deposition of amorphous silicate species to facilitate crystal growth. This observation indicates that the thermodynamically metastable amorphous precursor phase is converted into the stable zeolite crystalline phase. Notably, crystal growth accelerated drastically over the subsequent 6 h, producing aggregated nanocrystals with diminished amorphous residues (Figure 1d). This phenomenon points to a nonclassical crystallization mechanism for ZSM-5 fabricated via nanoseed induction without extra OSDAs [24,25]. This is further confirmed by the appearance of MFI diffraction peaks and a notable increase in relative crystallinity from 33% to 86% (Figure S2). This indicates that the MFI framework with long-range ordered three-dimensional intersecting straight and sinusoidal channels has largely formed. For an extended time of 6 h, a well-defined aggregate nanocrystal morphology was fully formed (Figure 1e), signifying the completion of the crystallization process. The aggregated nanocrystals exhibit an overall particle size of 330 nm, and the primary building units feature a *b*-axis thickness of 20 nm.

In summary, synthesizing ZSM-5 zeolite at 120 °C requires the system to undergo a long crystallization induction period of up to 60 h to complete the dissolution of the externally added seed crystals and the accumulation of nuclear fragments. Yet once the energy barrier is overcome (around 66 h), crystal growth advances rapidly and matures within 72 h.

**Figure 2:**
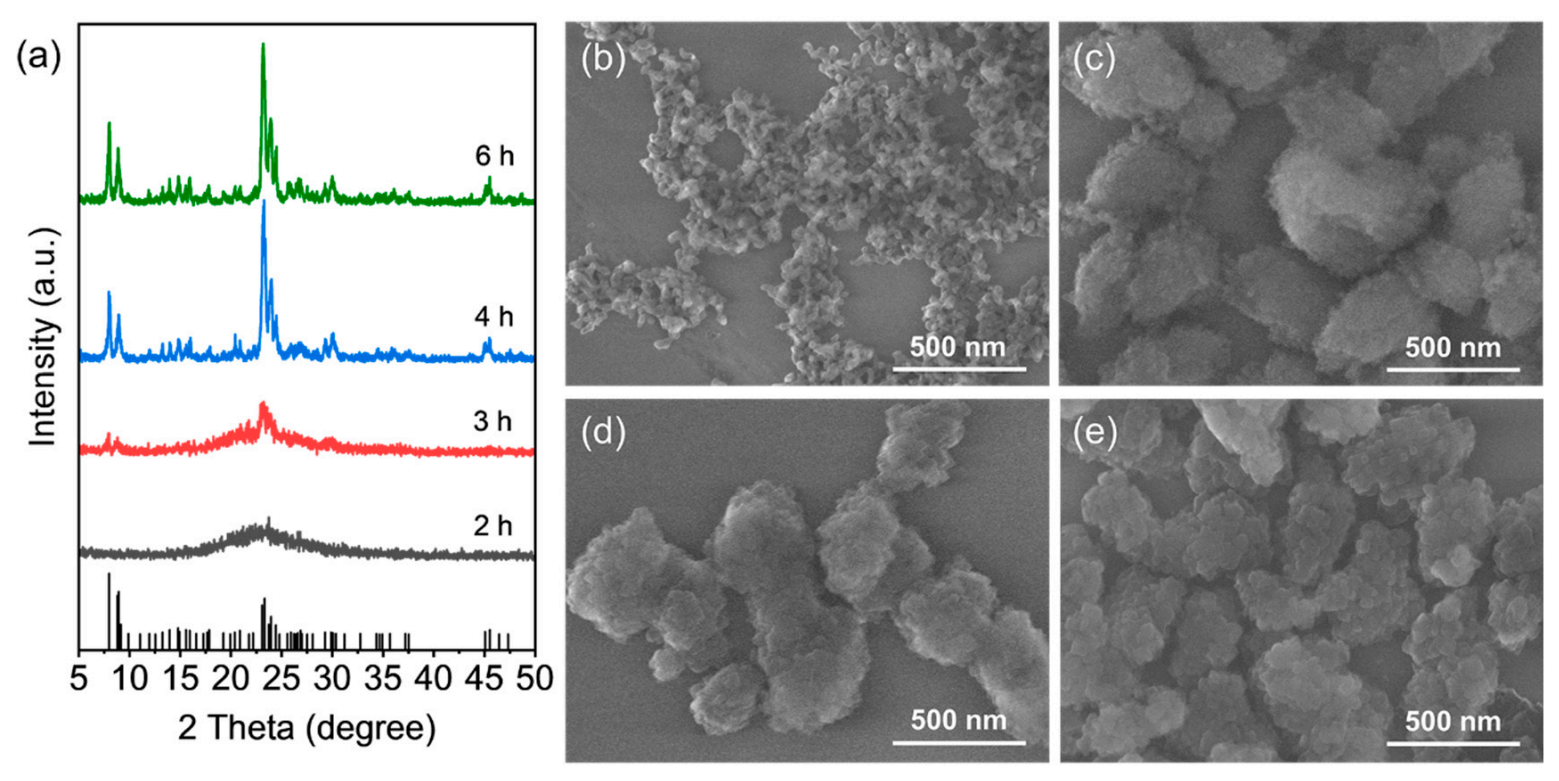
Characterization of ZSM-5 (170 °C) collected at various crystallization times. (**a**) XRD patterns. SEM images: (**b**) 2 h, (**c**) 3 h, (**d**) 4 h, and (**e**) 6 h.

The hydrothermal synthesis at 170 °C also undergoes an initial amorphous induction stage, yet the duration of this stage is drastically shortened. As it can be seen from Figure 2a that high-crystallinity and pure-phase ZSM-5 zeolites can be obtained within merely 4 h. In stark contrast to the 72 h of crystallization time required at 120 °C, this result highlights greatly improved synthesis efficiency. Meanwhile, Figure 2b–e visualize the nonclassical growth process whereby amorphous substances convert into aggregated crystals. Notably, although the product morphologically retains the characteristic of polycrystalline aggregation, the primary crystallites constituting these aggregates are visibly coarsened, and the assembled packing structure becomes much denser.

**Figure 3:**
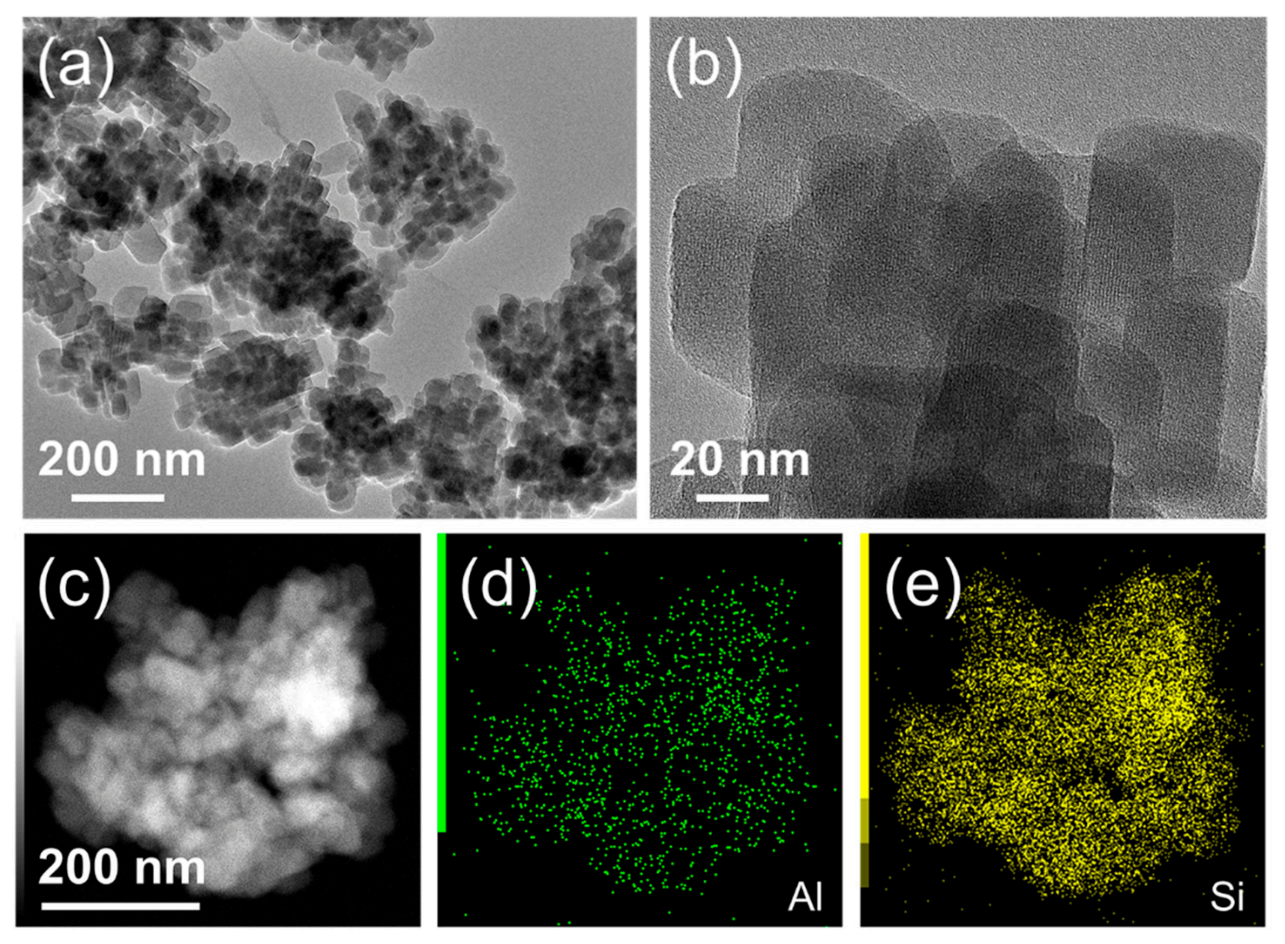
Microscopy characterization of the ZSM-5 (170 °C) sample. (**a**) Low-magnification and (**b**) high-magnification TEM images; (**c**) HAADF-STEM image of particle aggregates; (**d**) EDS mapping of Al distribution in the aggregates; (**e**) EDS mapping of Si distribution in the aggregates.

Figure 3 presents the transmission electron microscopy (TEM) and scanning transmission electron microscopy coupled with energy dispersive X-ray spectroscopy (STEM-EDS) mapping of the representative ZSM-5 (170 °C) sample crystallized at 6 h. As shown in Figure 3a,b, the TEM images reveal that the sample consists of uniform, well-defined nanocrystalline aggregates with clear intercrystalline boundaries, consistent with the stacking morphology observed by SEM. The lattice fringes between adjacent primary nanocrystallites exhibit consistent orientation along the *c*-axis, confirming that the primary crystallites are stacked in an oriented manner rather than randomly aggregated. Moreover, the corresponding STEM-EDS elemental maps (Figure 3c–e) show that Al is uniformly distributed throughout the crystallites, with no significant enrichment at the rim or any specific regions.

**Figure 4:**
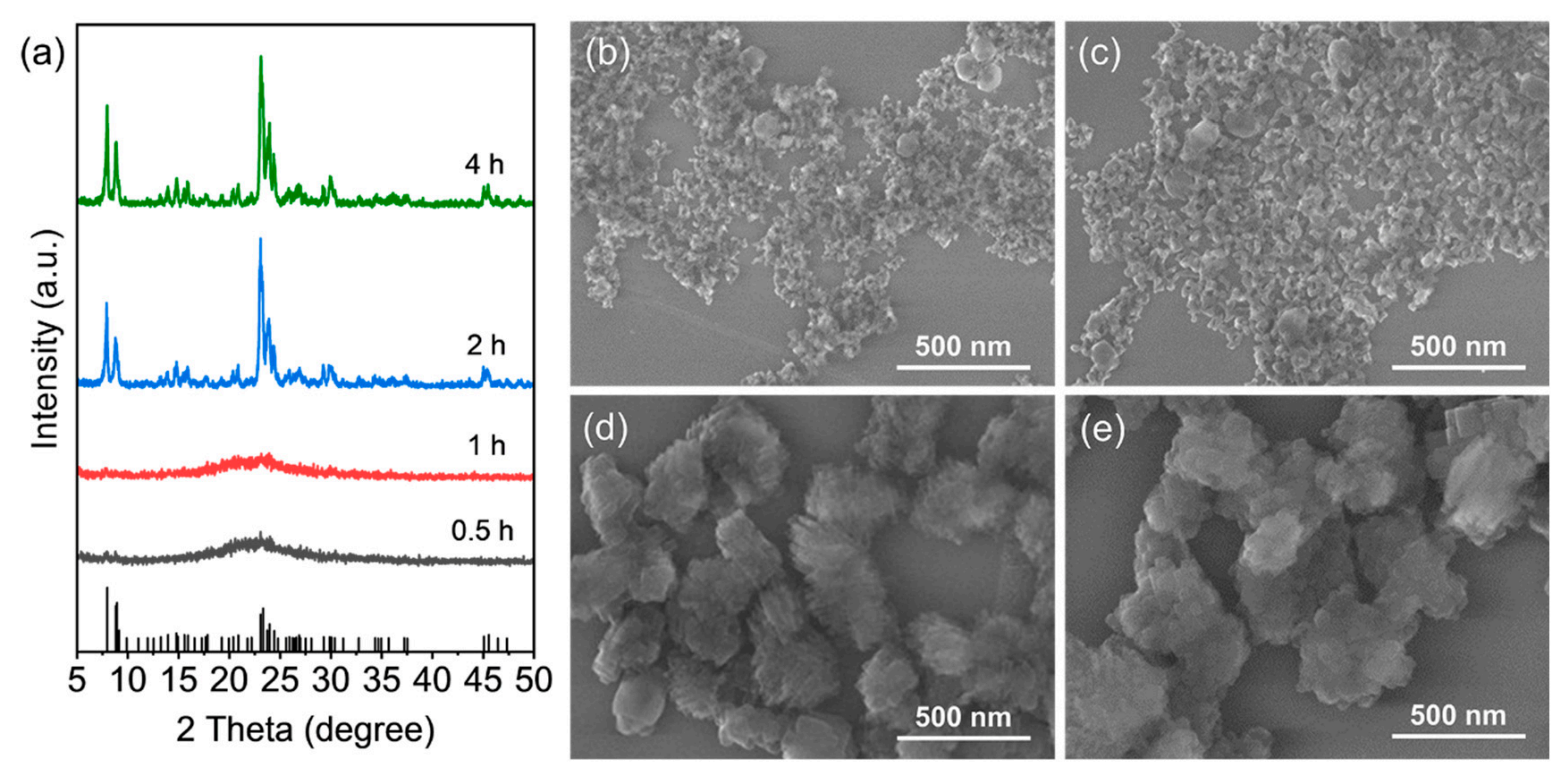
Characterization of ZSM-5 (200 °C) collected at various crystallization times. (**a**) XRD patterns. SEM images: (**b**) 0.5 h, (**c**) 1 h, (**d**) 2 h, and (**e**) 4 h.

Likewise, driven by the higher thermal energy at 200 °C, the precursor gel fully crystallizes within 2 h (Figure 4). In addition, even after extending the crystallization time to 4 h, no decline in crystallinity or phase transformation into thermodynamically dense impurity phases (e.g., quartz, cristobalite) is detected. This observation verifies that the constructed MFI framework possesses outstanding thermodynamic stability in extreme high-temperature hydrothermal media. Furthermore, Figure 4b–e show that the ZSM-5 crystals develop into aggregate nanocrystal morphologies composed of coarsened primary grains. These crystals display uniform particle sizes, with all amorphous surface residues completely eliminated.

To provide an overall comparison of the crystallization kinetics, the time for complete crystallization at each temperature is summarized in Figure S3. As clearly shown, increasing the crystallization temperature from 120 °C to 170 °C and 200 °C drastically shortens the crystallization time from 72 h to 4 h and 2 h, respectively, highlighting the significant acceleration effect of higher thermal energy on the crystal growth process, especially within the lower temperature range of 120–170 °C.

These results reveal that the increased crystallization temperature does not alter the inherent nonclassical growth mechanism of MFI crystals induced by seed crystals. The main difference among samples lies in the coarsening of primary crystallite units as the temperature rises. This phenomenon originates from the strong thermal driving force at high temperature, which accelerates the anisotropic growth rate of the crystals and weakens the originally prominent thermodynamic growth preference along the *c*-axis relative to the *a*-axis and *b*-axis. Meanwhile, the mass transfer rate at the liquid–solid interface is significantly increased, speeding up the directional migration and epitaxial deposition of silicate and aluminate monomers toward crystal faces. These two effects jointly result in the primary crystallite units no longer presenting as small nanoplates but as equiaxed nanoparticles, thereby overall presenting a coarsened aggregate morphology. Macroscopically, this disordered loose packing morphology leaves irregular voids at the splicing seams between nanocrystals, thereby spontaneously deriving abundant intercrystalline mesopores.

### 2.2. Physicochemical Properties of ZSM-5 Synthesized at Different Temperatures

These samples, collected at 72 h, 6 h, and 4 h for 120, 170, and 200 °C, respectively, have been thoroughly characterized with regard to their elemental composition, textural and acid properties.

**Figure 5:**
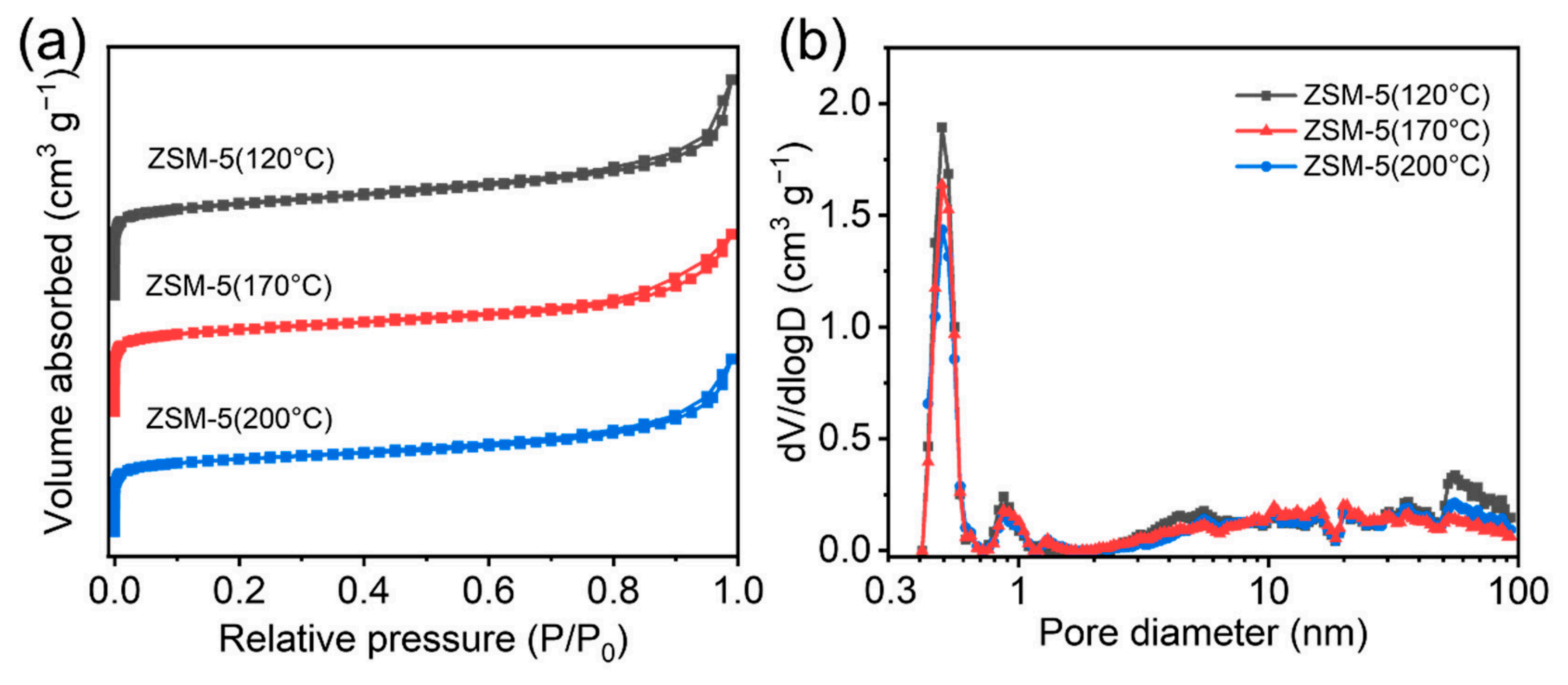
Characterization of ZSM-5 samples synthesized at different temperatures. (**a**) Ar adsorption–desorption isotherms and (**b**) the corresponding DFT pore size distributions.

Figure 5 shows the Ar adsorption–desorption isotherms of the as-prepared ZSM-5 zeolites. All curves conform to type I physisorption behavior typical of microporous materials, with a steep increase in the gas adsorbed at low relative pressures [26]. Corresponding textural properties determined from the isotherms are listed in Table 1. Overall, the specific surface area and pore volume indicators of the materials show a volcano-shaped variation with the crystallization temperature. As the temperature rises from 120 °C to 170 °C and further to 200 °C, the total specific surface area first increases from 344 m^2^ g^−1^ to a maximum of 427 m^2^ g^−1^ before declining to 384 m^2^ g^−1^. Similarly, the microporous surface area expands from 268 m^2^ g^−1^ to 323 m^2^ g^−1^, while the micropore volume reflecting the intrinsic framework structure increases from 0.13 cm^3^ g^−1^ to 0.16 cm^3^ g^−1^. Such variations can be mainly ascribed to differences in the crystallinity and crystal morphology across samples. The weak peak intensity of ZSM-5 (120 °C) sample shown in Figure S4 further supports this. Elevated temperatures facilitate the full development of the three-dimensional pore structure by clearing out amorphous residues that could block the intrinsic channels and eliminating the defects that affect the framework stability. The pore size distribution curves shown in Figure 5b reveal abundant intercrystalline mesopores ranging from 2 nm to 50 nm, consistent with the slit-like voids generated by the irregular stacking of primary nanocrystals observed in the SEM images. The simultaneous increase in specific surface area and integrated micro-mesopore volume greatly enhances the accessibility of active sites and accelerates the diffusion of bulky molecules [8,27].

The acid properties (concentration and intrinsic strength) of ZSM-5 zeolites are one of the key factors determining their catalytic performance. Variations in crystallization temperature directly alter the coordination state and occupancy positions of Al atoms throughout crystal growth, which further tunes the overall acid characteristics.

**Figure 6:**
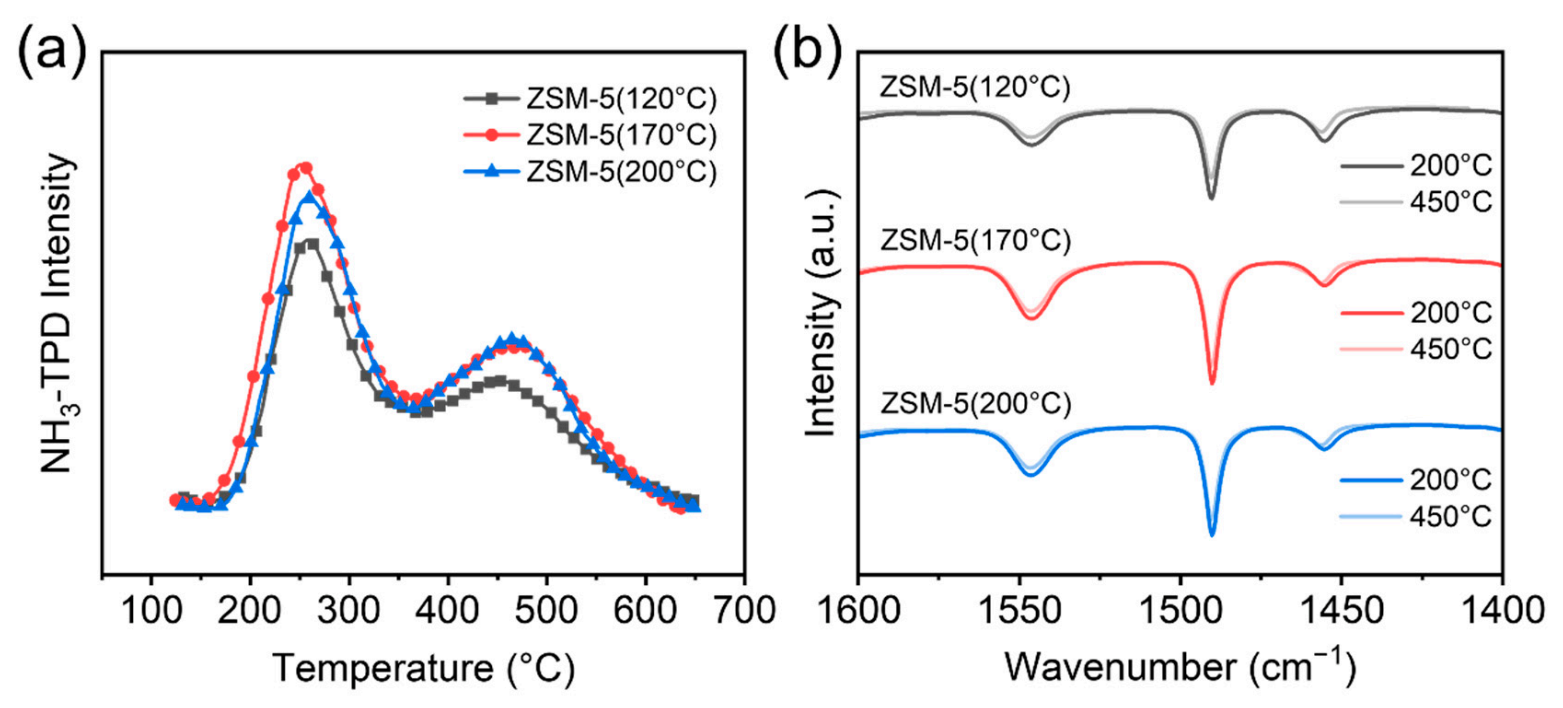
Characterization of ZSM-5 samples synthesized at different temperatures. (**a**) NH_3_-TPD curves and (**b**) Py-IR spectra.

Inductively coupled plasma optical emission spectroscopy (ICP-OES), ammonia temperature-programmed desorption (NH_3_-TPD) and pyridine adsorption infrared spectroscopy (Py-IR) were employed to systematically investigate the differences in acid properties among the prepared ZSM-5 samples. Figure 6 shows the corresponding NH_3_-TPD curves and Py-IR spectra. It can be seen from Figure 6a that all samples display two well-resolved NH_3_ desorption peaks centered at ~250 °C and ~470 °C. The low-temperature peak corresponds to weak acid sites, whereas the high-temperature peak originates from strong acid sites within the zeolite framework [28]. Quantitative acid parameters derived from peak deconvolution are listed in Table 2. As seen, the ZSM-5 (120 °C) sample features the lowest concentrations of weak and strong acid sites, at 334 μmol g^−1^ and 242 μmol g^−1,^ respectively. This result matches its high bulk SiO_2_/Al_2_O_3_ molar ratio of 68 measured by ICP-OES, which can be explained by the low crystallinity at 120 °C that results in a fraction of Al species failing to enter the zeolite framework.

In comparison, ZSM-5 (170 °C) and ZSM-5 (200 °C) possess SiO_2_/Al_2_O_3_ molar ratios close to the bulk phase value of 40, and both bear abundant acid sites. Of these two samples, ZSM-5 (170 °C) exhibits a slightly higher acid concentration. Its weak acid concentration reaches 471 μmol g^−1^, and the strong acid concentration also expands to 339 μmol g^−1^. The coordination state of Al species in the ZSM-5 (170 °C) sample was further investigated by solid-state ^27^Al MAS NMR spectroscopy. As shown in Figure S5, the spectrum displays a dominant resonance at ~54 ppm, corresponding to tetrahedrally coordinated framework Al, along with a weak signal at ~0 ppm attributable to a minor amount of octahedral extra-framework Al species. This result confirms that the elevated crystallization temperature facilitates the incorporation of Al atoms into framework tetrahedral T-sites.

The Py-IR spectra shown in Figure 6b distinguish between the Brønsted acid sites (BAS, originating from framework bridging hydroxyl groups) at 1546 cm^−1^ and Lewis acid sites (LAS) at 1450 cm^−1^ [29,30,31]. Py-IR measurements were collected at 200 °C and 450 °C for each sample, allowing quantitative calculation of BAS and LAS concentrations with distinct acid strengths. The detailed calculation method is provided in the Supporting Information. As seen in Table 2, the absolute acid concentrations determined by Py-IR are systematically lower than those measured by NH_3_-TPD, which is attributable to the smaller molecular size of NH_3_ and its non-selective adsorption on various sites, while pyridine selectively probes Brønsted and Lewis acid sites. The BAS concentration measured at 200 °C desorption rises sharply for the ZSM-5 (170 °C) and ZSM-5 (200 °C) samples, reaching 297 μmol g^−1^ and 273 μmol g^−1^, respectively, far exceeding the BAS concentration measured for ZSM-5 (120 °C) (only 192 μmol g^−1^). However, the LAS concentration shows a decreasing trend as crystallization temperature increases. These observations indicate that crystallization temperature exerts a regulatory effect on the concentration of BAS and LAS. Notably, with the increase of desorption temperature to 450 °C, the majority of both BAS and LAS remain, indicating that most acid sites belong to strong acids.

Collectively, in a seed-assisted low-template system, high hydrothermal temperatures facilitate the formation of highly crystalline ZSM-5 and accelerate the entry of Al species into the zeolite framework. This is a key factor leading to the improvement of its textural properties and acid concentration. Given that a high concentration of strong BAS serves as the core active sites to polarize benzene rings and protonate methanol for subsequent electrophilic substitution, these tailored acid properties formulated by temperature regulation directly affect the catalytic behavior of these ZSM-5 samples in benzene–methanol alkylation.

### 2.3. Catalytic Performance of ZSM-5 Synthesized at Different Temperatures in Benzene Alkylation with Methanol

These three catalysts were evaluated for benzene alkylation with methanol to reveal how distinct textural properties and acid concentrations jointly govern catalytic behavior. Benzene–methanol alkylation proceeds through complex competing reaction pathways. Previous literature has proven that even minor differences in the microstructure and acidity readily trigger severe coke deposition blockage within the channels, leading to rapid catalyst deactivation and loss of long-term operational stability [9,10,11,32,33].

**Figure 7:**
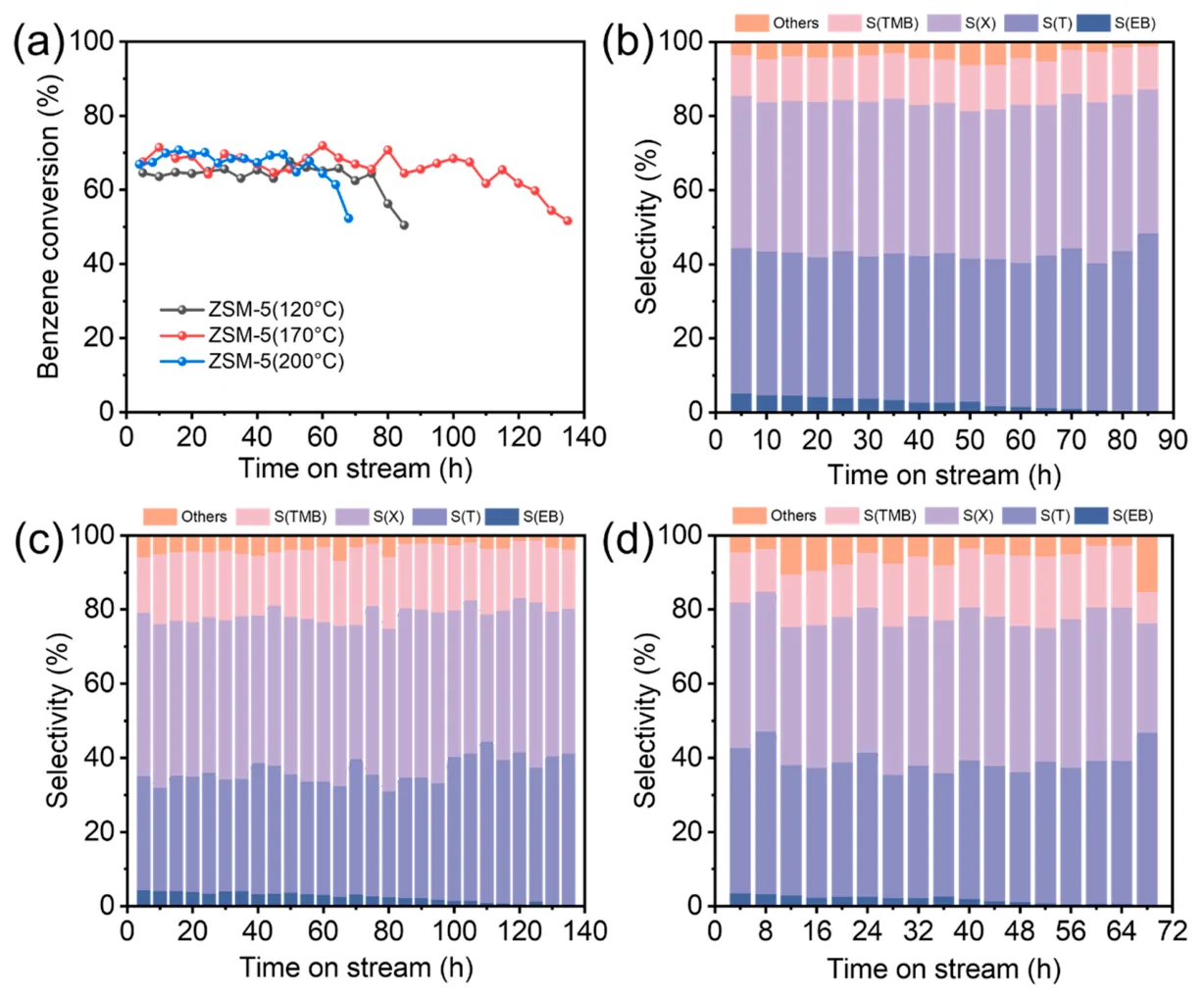
Benzene conversion and aromatic selectivity over ZSM-5 samples synthesized at different temperatures as a function of time on stream. (**a**) Benzene conversion. Aromatic selectivity: (**b**) ZSM-5 (120 °C), (**c**) ZSM-5 (170 °C), and (**d**) ZSM-5 (200 °C) sample. Reaction conditions: 0.5 g of catalyst, 460 °C, WHSV = 4 h^−1^, atmospheric pressure, and the molar ratio of benzene to methanol is 1:1.5. Note: TMB, X, T, and EB stand for trimethylbenzene, xylene, toluene, and ethylbenzene, respectively.

Figure 7 depicts the evolution of benzene conversion and product selectivity (toluene, xylene, trimethylbenzene and ethylbenzene byproduct) as functions of time on stream (TOS). Benzene conversion remains stable at the initial stage before dropping sharply with prolonged TOS. In contrast, ethylbenzene selectivity decreases monotonically, while toluene selectivity rises moderately. Such variations originate from the progressive buildup of coke deposits on Brønsted acid sites. At early TOS, mild coke coverage suppresses the methanol-to-olefin (MTO) pathway and ethylene generation, thus limiting byproduct ethylbenzene production [8]. As the reaction proceeds, extensive coke accumulation gradually covers most acid sites and ultimately induces rapid catalyst deactivation.

The reaction results at TOS of 20 h are summarized in more detail in Table 3. Differences in catalytic activity, target aromatic selectivity, methanol utilization and catalyst lifetime are evident across the three catalysts. Among them, the ZSM-5 (170 °C) sample exhibits the optimal overall performance, followed by ZSM-5 (200 °C), while ZSM-5 (120 °C) was the worst. Specifically, the ZSM-5 (170 °C) sample achieves a high benzene conversion of 69.1%, a high methanol utilization of 79.1%, and a long lifetime of 135 h. Compared with the other two samples, although the catalytic lifetime of ZSM-5 (200 °C) is slightly shorter than that of ZSM-5 (120 °C), all other indicators demonstrate comprehensive superiority. The significant disparities in catalytic performance can be attributed to their distinct textural structures and acid properties, namely, specific surface area, mesopore volume, and BAS acid concentration (the key active sites for benzene alkylation). The corresponding explanations are as follows: (i) Larger total specific surface area (344 vs. 427 vs. 384 m^2^ g^−1^, for 120, 170 and 200 °C, respectively) strengthens contact between reactants and active sites, raising benzene conversion and the selectivity of consecutive alkylation products (xylene and trimethylbenzene). Meanwhile, abundant surface sites mitigate rapid coking and extend catalyst lifetime; (ii) Larger mesopore volume formed by the polycrystalline stacking (0.18 vs. 0.23 vs. 0.17 cm^3^ g^−1^) facilitates mass transport of bulky aromatic intermediates, suppressing the accumulation of coke precursors inside micropores. Meanwhile, it also accommodates more coke deposits, further prolonging the lifetime; (iii) Higher BAS concentration (192 vs. 297 vs. 273 μmol·g^−1^) offers abundant active sites, enabling efficient adsorption of methanol and catalyzing its dehydration to form active carbocationic species, thereby improving the benzene conversion and consecutive alkylation products.

Overall, by rationally regulating the BAS acid concentrations and textural structures via tuning the crystallization temperature, ZSM-5 (170 °C) achieves an optimal trade-off among conversion, target aromatic selectivity, methanol utilization and long-term stability.

**Figure 8:**
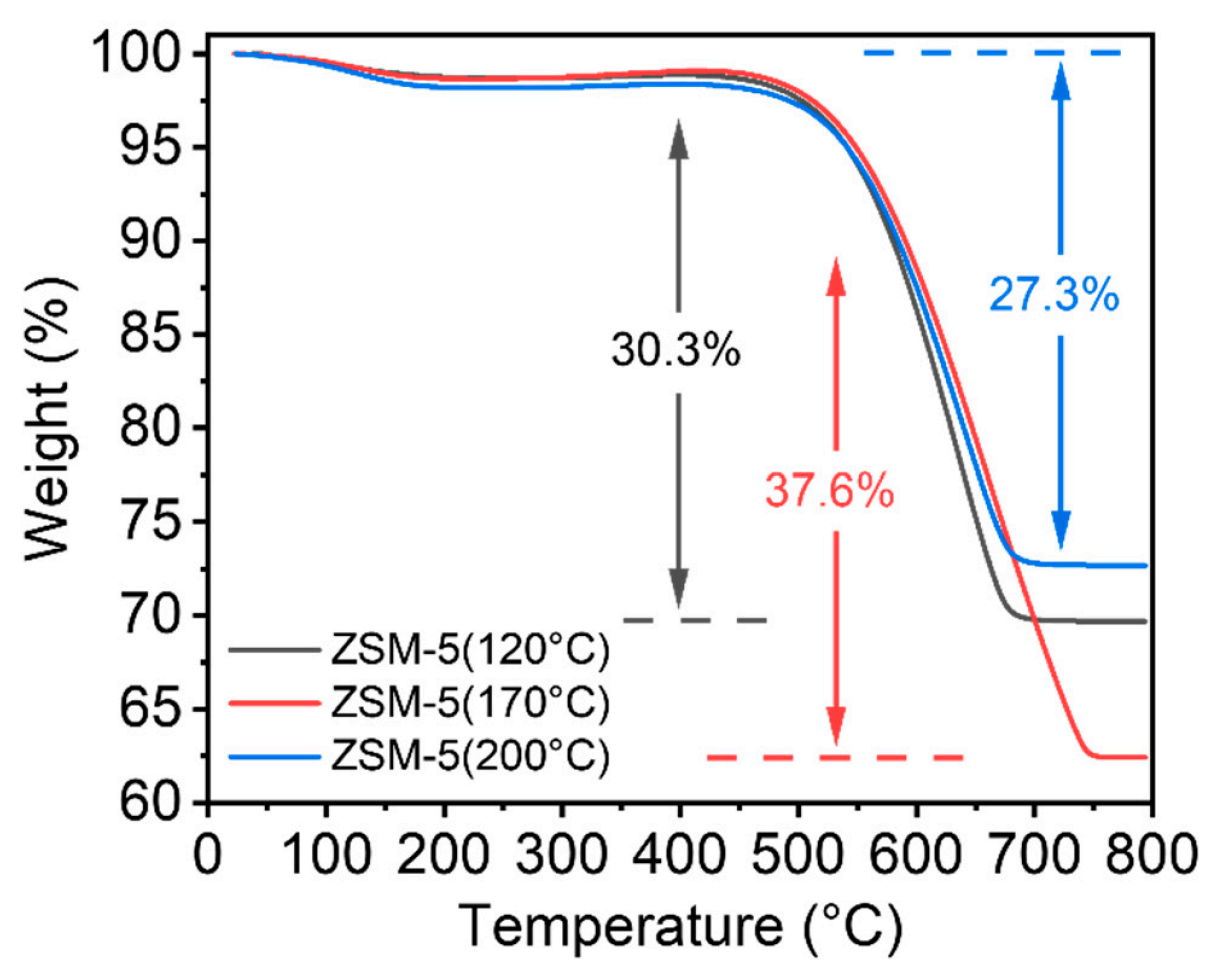
TG curves of spent ZSM-5 catalysts.

Thermogravimetric (TG) measurements were performed on spent catalysts for probing the content and degree of the coke species. As illustrated in Figure 8, the weight loss below 200 °C is primarily attributed to the desorption of physically adsorbed substances, including water, unreacted benzene and aromatic products. The weight loss above 400 °C corresponds to the thermal decomposition and combustion of coke deposits [9]. Quantitative coke parameters are summarized in Table 4. Compared with ZSM-5 (120 °C) and ZSM-5 (200 °C), the ZSM-5 (170 °C) sample accumulates the highest coke content (303 vs. 376 vs. 273 mg·g_cat_^−1^) yet exhibits the lowest coking rate (3.56 vs. 2.79 vs. 4.01 mg·g_cat_^−1^·h^−1^) during long-term operation. This confirms that the well-matched textural and acid characteristics of the ZSM-5 (170 °C) sample effectively suppress coke formation and allow the catalyst to tolerate more coke deposits, endowing it with excellent anti-deactivation performance.

## 3. Materials and Methods

### 3.1. Chemicals

The synthesis of ZSM-5 involves the following chemicals: tetrapropylammonium hydroxide (TPAOH, 25 wt%, Shanghai Cainorise Chemicals Co., Ltd., Shanghai, China), tetraethoxysilane (TEOS, >98%, Xilong Scientific Co., Ltd., Shantou, China), ethanol (CH_3_CH_2_OH, ≥99.8%, Tianjin Fuyu Fine Chemical Co., Ltd., Tianjin, China), sodium aluminate (NaAlO_2_, >97%, Shanghai Aladdin Biochemical Technology Co., Ltd., Shanghai, China), silica sol (SiO_2_, 30 wt%, Shanghai Aladdin Biochemical Technology Co., Ltd., Shanghai, China), ammonium chloride (NH_4_Cl, ≥99.5%, Tianjin Damao Chemical Reagent Factory, Tianjin, China), and sodium hydroxide (NaOH, ≥97%, Tianjin Damao Chemical Reagent Factory, Tianjin, China).

### 3.2. Synthesis of Silicalite-1 Seed Suspension

Silicalite-1 seed suspension was prepared under hydrothermal treatment with the molar composition of 1 TEOS: 0.35 TPAOH: 14.4 H_2_O: 7.9 EtOH. Typically, 21.70 g of TEOS, 29.66 g of TPAOH (25 wt%), 4.76 g H_2_O and 18.69 g EtOH were mixed and stirred for 2 h at room temperature to obtain a hydrolyzed clear solution. Then, the solution was transferred into a Teflon-lined stainless-steel autoclave and hydrothermally treated at 120 °C under static conditions for 48 h to obtain a silicalite-1 seed suspension with a particle size of 100 nm (Figure S6).

### 3.3. Low-Template Synthesis of ZSM-5 Zeolites

The term “low-template” mentioned in this work indicates that the synthetic gel contains only trace residual template (TPA^+^/SiO_2_ = 0.018, with respect to the SiO_2_ in the silica sol) originating from the liquid silicalite-1 seed suspension, without additional organic structure-directing agents added.

The synthesis gel composition was 1 SiO_2_: 0.025 Al_2_O_3_: 50 H_2_O: 0.21 NaOH: 5 wt% seed suspension. Firstly, 0.365 g NaAlO_2_, 0.431 g NaOH and 15.00 g silica sol were dissolved in 57.00 g deionized water as the synthesis gel. After stirring for 1 h, 2.72 g of silicalite-1 seed suspension was added and then stirred for another 20 min to get the precursor gel. Then the gel was transferred into a Teflon-lined stainless-steel autoclave, and crystallized at 90 °C, 120 °C, 170 °C and 200 °C under rotation (30 r·min^−1^) in a homogeneous reactor. The solid product was collected, centrifuged with deionized water, and dried at 80 °C to obtain the Na-ZSM-5 sample. Subsequently, the obtained Na-ZSM-5 zeolites were turned into H-form by ion exchange with NH_4_Cl solution (1.0 mol·L^−1^) at 80 °C for 2 h with a liquid/solid ratio of 20 g·g^−1^. The process was repeated three times. After that, the NH_4_-ZSM-5 sample was centrifuged, washed, dried, and then calcined at 540 °C for 4 h. The resulting H-ZSM-5 products were denoted as ZSM-5 (x), where x represents the crystallization temperature. The yields for ZSM-5 (120 °C), ZSM-5 (170 °C), and ZSM-5 (200 °C) are 76%, 93%, and 90%, respectively.

Following a procedure similar to that described above, the time-dependent crystallization studies were conducted for each designated time point using separate autoclaves under identical conditions.

### 3.4. Characterization

X-ray diffraction (XRD) was performed on a Rigaku SmartLab 9 kw diffractometer with a Cu Kα (λ = 1.5406 Å) radiation source. Measurement was conducted at a scanning rate of 8° min^−1^. All samples were in powder form weighing 100 mg exactly for diffraction intensity comparison. The relative crystallinity was calculated by comparing the diffraction intensities of the five typical characteristic peaks of the ZSM-5 zeolite at 2θ = 7.9, 8.8, 23.1, 23.9, and 24.5° to the sample with the highest intensity.

Scanning electron microscopy (SEM) was conducted using the SU8200 (Hitachi) electron microscope with an accelerating voltage of 5 kV. The average particle size was obtained by measuring more than 100 particles.

Transmission electron microscopy (TEM), high-angle annular dark-field scanning transmission electron microscopy (HAADF-STEM) and energy dispersive X-ray spectroscopy (EDS) analyses were performed on F200 (JEOL) with accelerating voltages of 200 kV. The powder samples were dispersed in ethanol and then several drops of the suspension were dripped on carbon films.

^27^Al MAS NMR spectrum was recorded on a Bruker Avance 500 MHZ spectrometer at a spinning rate of 15 kHz and a pulse length of 2.3 μs. Al(NO_3_)_3_ (1.0 M) was used as the reference (at 0 ppm) for the chemical shifts of ^27^Al.

Ar adsorption-desorption isotherms of ZSM-5 samples were measured at −186 °C using an Autosorb-iQ gas adsorption analyzer (Quantachrome, Boynton Beach, FL, USA). Before measurements, samples were heated at 300 °C for 8 h in vacuum for degassing.

Ammonia temperature-programmed desorption (NH_3_-TPD) was analyzed on a ChemBET Pulsar TPR equipment (Quantachrome, USA) in a quartz tube reaction vessel. 0.1 g of sample was placed at the bottom of tube and degassed at 500 °C for 60 min, then cooled to 120 °C for NH_3_ adsorption. After removing physisorbed NH_3_, NH_3_-TPD data were recorded from 120 °C to 650 °C with a ramp of 10 °C min^−1^.

The concentration of Brønsted and Lewis acid sites of ZSM-5 samples was measured by pyridine adsorption experiments (Py-IR) using a Thermo Scientific Nicolet iS50 Fourier transform infrared spectrometer (Thermo Fisher Scientific Inc., Waltham, MA, USA). Before adsorption test, 20 mg of sample was pressed into a self-supporting wafer (14 mm i.d.) and loaded on the IR cell. After pretreatment under vacuum at 450 °C for 60 min, it was cooled to room temperature to record the background spectrum, and then pyridine molecules were introduced. Next, the cell was evacuated at 200 °C and 450 °C for 35 min, and cooled to room temperature to record the spectra, respectively. The Brønsted and Lewis acid sites were identified from the IR bands at 1546 and 1456 cm^−1^, respectively, and the concentration was calculated via the Beer–Lambert law using the following equation [34]:

$$C\;=\;X\;\times \;\frac{AR^{2}}{m}\;\times \;1000$$

where C is the concentration of acid sites (μmol g^−1^), A is the area of the IR band (cm^−1^), R is the radius of the self-supporting wafer (cm), the X values are 1.88 and 1.42 for Brønsted and Lewis acid sites, respectively, and m is the loaded sample mass (mg). The total Brønsted and Lewis acid concentration is quantified by the Py-IR curves obtained at 200 °C and the strong acid concentration is quantified by the Py-IR curves obtained at 450 °C.

The elemental compositions were analyzed by inductively coupled plasma optical emission spectroscopy (ICP-OES) using a PerkinElmer AVIO 500 unit.

Thermogravimetric analysis (TGA) of coke content over spent samples was performed using a SDTQ-600 analyzer from room temperature to 800 °C with a ramp of 10 °C min^−1^ under an air flow of 100 mL min^−1^.

### 3.5. Catalytic Tests

Alkylation of benzene with methanol was carried out in a fixed-bed reactor at atmospheric pressure and 460 °C. Typically, 0.5 g of catalyst (20–40 mesh) was mixed with 1.5 g of quartz sand (20–40 mesh) and then loaded in the middle of a quartz tube. The molar ratio of benzene to methanol in the feed was 1:1.5, and the total weight hour space velocity (WHSV) was maintained at 4 h^−1^. Before the reaction, the catalyst was heated up to 460 °C with a ramp rate of 5 °C·min^−1^ in pure N_2_ flow (30 mL·min^−1^). The aromatic products were analyzed using a gas chromatograph (GC9720, FULI Co., Ltd., Taizhou, China) with a flame ionization detector (FID) and a PEG-20M capillary column (50 m × 0.32 mm × 0.5 μm). The benzene conversion (X), the selectivity of aromatic products (S*_i_*) and methanol utilization (U_m_) were calculated as follows:

$$X\;(\%)=\frac{n_{benzene,in}\;-\;n_{benzene,out}}{n_{benzene,in}}\times 100\%$$

$$S_{i}\;(\%)=\frac{n_{i}}{{\sum n}_{i}}\times 100\%$$

$$U_{m}\;(\%)=\frac{n_{Methanol\;in\;target\;aromatic\;products}}{n_{Methanol\;in\;products}}\times 100\%$$

Here, n_i_ is an aromatic product, and the target aromatic products are toluene, xylene, and trimethylbenzene.

## 4. Conclusions

This work systematically investigates how gradient crystallization temperatures regulate the microstructural evolution of seed-assisted low-template ZSM-5 zeolites, and further establishes the underlying structure–activity relationship for benzene alkylation with methanol. The specific findings may be summarized below.

(1) A seed-assisted low-template strategy is developed to synthesize ZSM-5 nanoaggregates, whose formation proceeds via a nonclassical crystallization mechanism.
(2) Elevated crystallization temperature provides sufficient thermal energy for strongly alkaline gel systems to overcome heterogeneous crystallization energy barriers. A nearly 60 h amorphous induction period exists at 120 °C with slow structural rearrangement of precursor. By contrast, when the temperature rises to 200 °C, amorphous gel transforms into a highly crystalline MFI structure within only 2 h.
(3) Elevating crystallization temperature transforms crystal morphology from stacked nanosheet aggregates to densely packed nanoparticle assemblies, which generates a large specific surface area and abundant intercrystalline mesopores. Meanwhile, the high-temperature hydrothermal environment forces Al species to embed into tetrahedral framework T-sites, thereby significantly improving the BAS concentration.
(4) For benzene–methanol alkylation, either insufficient or excessively high crystallization temperature impairs overall catalytic performance. The ZSM-5 catalyst synthesized at 170 °C delivers the optimal trade-off between catalytic activity and anti-deactivation performance, achieving a benzene conversion of 69.1%, methanol utilization of 79.1%, and a long lifetime of 135 h. Such superior catalytic behavior originates from its optimized combination of large specific surface area, abundant intercrystalline mesopores and BAS acid concentration, which synergistically accelerate mass transfer of aromatic intermediates and suppress rapid coke accumulation.

## Figures and Tables

**Table 1:** Texture properties of ZSM-5 samples synthesized at different temperatures.

Samples	Surface Area ^a^ (m^2^ g^−1^)			Pore Volume ^b^ (cm^3^ g^−1^)		
	Total	Micro.	Ext.	Total	Micro.	Meso.
ZSM-5 (120 °C)	344	268	76	0.31	0.13	0.18
ZSM-5 (170 °C)	427	323	104	0.39	0.16	0.23
ZSM-5 (200 °C)	384	306	78	0.32	0.15	0.17

^a^ Determined via the BET method (S_Total_) and t-plot method (S_Micro._ and S_Ext._). ^b^ Taken as the Ar adsorption volume at P/P_0_ = 0.99 (V_Total_) and determined via the t-plot method (V_Micro._).

**Table 2:** Acid properties of ZSM-5 samples synthesized at different temperatures.

Sample	SAR ^a^	Acid Concentration (μmol·g^−1^)					
		Weak ^b^	Strong ^b^	BAS ^c^		LAS ^c^	
				200 °C	450 °C	200 °C	450 °C
ZSM-5 (120 °C)	68	334	242	192	150	91	60
ZSM-5 (170 °C)	48	471	339	297	247	65	52
ZSM-5 (200 °C)	54	432	314	273	235	70	54

^a^ Determined by ICP-OES. ^b^ Calculated by deconvolution of the NH_3_-TPD curves. ^c^ Calculated by deconvolution of the Pyridine-IR curves.

**Table 3:** Benzene conversion and aromatic selectivity over ZSM-5 samples synthesized at different temperatures at TOS of 20 h ^a^.

Sample	Conv. (%)	Aromatic Selectivity (%)					U_m_ ^b^ (%)
		EB	Toluene	Xylene	TMB	Others	
ZSM-5 (120 °C)	64.4	4.2	37.7	41.9	12.0	4.2	67.6
ZSM-5 (170 °C)	69.1	3.9	31.0	41.7	19.1	4.3	79.1
ZSM-5 (200 °C)	69.7	2.6	36.3	39.2	14.2	7.7	73.1

^a^ Reaction conditions: 0.5 g of catalyst, 460 °C, WHSV = 4 h^−1^, atmospheric pressure, and the molar ratio of benzene to methanol is 1:1.5. ^b^ U_m_ denotes methanol utilization (see Section 3.5 for calculation).

**Table 4:** Coke deposition content and coking rate of spent ZSM-5 catalysts.

Samples	TOS (h)	Coke Deposition Content ^a^ (mg·g_cat_^−1^)	Coking Rate ^b^ (mg·g_cat_^−1^·h^−1^)
ZSM-5 (120 °C)	85	303	3.56
ZSM-5 (170 °C)	135	376	2.79
ZSM-5 (200 °C)	68	273	4.01

^a^ Calculated by weight loss in TG curves; ^b^ Calculated by the ratio of coke content to TOS.

## Data Availability

The original contributions presented in the study are included in the article (and Supplementary Materials), further inquiries can be directed to the corresponding authors.

## Supplementary Materials

The following supporting information can be downloaded at: https://www.mdpi.com/article/10.3390/molecules31173084/s1, Figure S1: XRD pattern of the ZSM-5 sample synthesized at 90 °C for 144 h; Figure S2: Relative crystallinity of ZSM-5 (120 °C) samples collected at various crystallization times; Figure S3: Dependence of the crystallization time on synthesis temperature for ZSM-5 zeolites. Figure S4: XRD patterns of the ZSM-5 samples synthesized at different crystallization temperatures; Figure S5: ^27^Al MAS NMR spectrum of the ZSM-5 (170 °C) sample. Figure S6: SEM image of silicalite-1 seed crystals.
