# Supplementary material for: Temperature-Dependent Microstructural Evolution of Seed-Assisted Low-Template ZSM-5 and Its Catalytic Behavior in Benzene Alkylation with Methanol
Source: Molecules. 2026 Sep 2;31(17):3084. doi: 10.3390/molecules31173084 (PMC13567218; doi:10.3390/molecules31173084)
Supplement: Supplementary file 1 [file molecules-31-03084-s001.zip › molecules-4472069-supplementary.pdf]

Supporting Information

Temperature-Dependent Microstructural Evolution of Seed-Assisted Low-Template ZSM-5 and Its Catalytic Behavior in Benzene Alkylation with Methanol

Shuyi Wu[a], *Yanji Shi*[a], *Qihao Zheng*[a], *Jianle Yuan*[b], *Yilin Xiang*[a], *Yining Jiang*[b], *Jiaxing Zhang*[a]*, *Ajuan Zhou*[c]*, *Xinwen Guo*[a,b]*

[a] School of Chemical Engineering, Ocean and Life Sciences, Dalian University of Technology, Panjin 124221, China

[b] State Key Laboratory of Fine Chemicals, Frontiers Science Center for Smart Materials, School of Chemical Engineering, Dalian University of Technology, Dalian 116024, China

[c] Liaoning Key Laboratory of Chemical Additive Synthesis and Separation, Yingkou Institute of Technology, Yingkou 115014, Liaoning Province, China;

Corresponding author. Email: zhangjx@dlut.edu.cn; zhouajuan@yku.edu.cn; guoxw@dlut.edu.cn

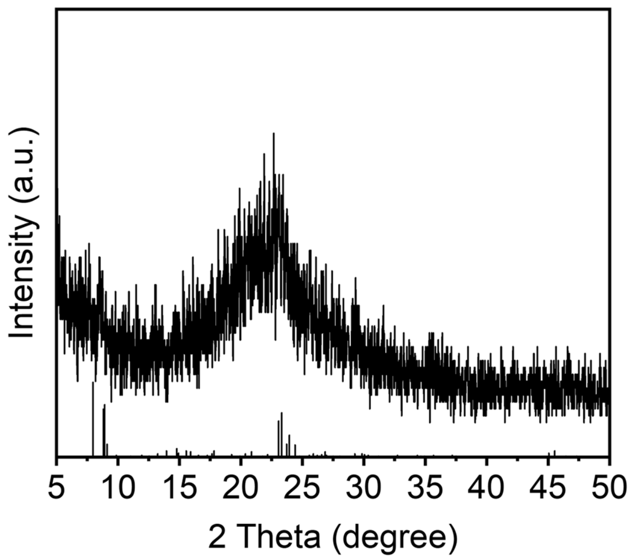

Figure S1. XRD pattern of the ZSM-5 sample synthesized at 90 °C for 144 h.

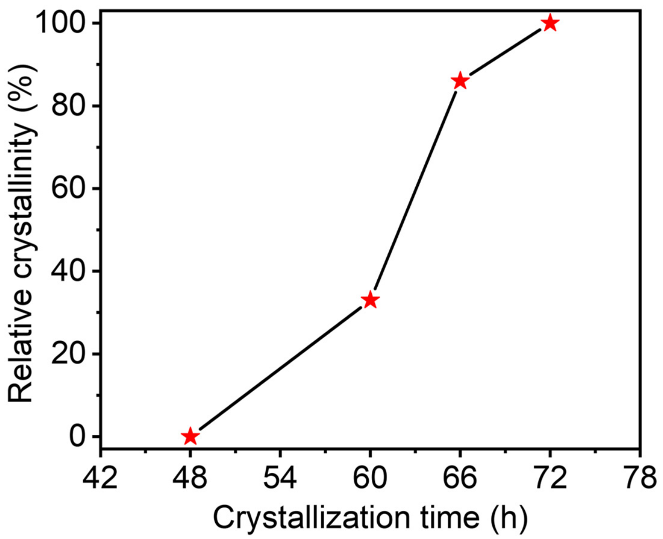

Figure S2. Relative crystallinity of ZSM-5(120°C) samples collected at various crystallization times.

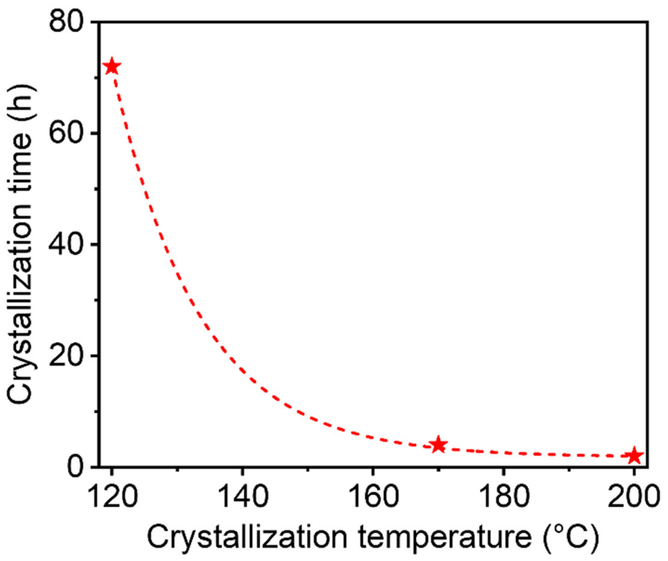

Figure S3. Dependence of the crystallization time on synthesis temperature for ZSM-5 zeolites.

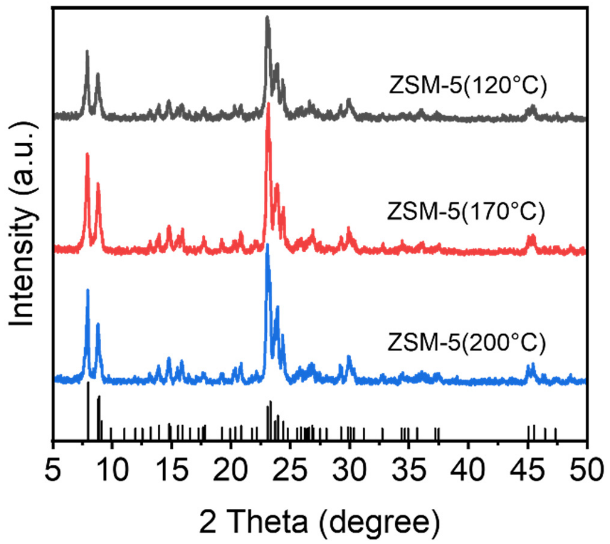

Figure S4. XRD patterns of the ZSM-5 samples synthesized at different crystallization temperatures.

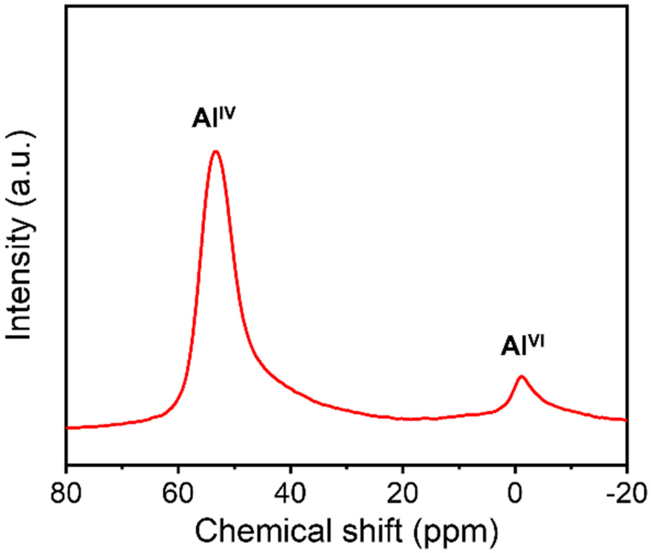

Figure S5. ^{27}Al MAS NMR spectrum of the ZSM-5(170 °C) sample.

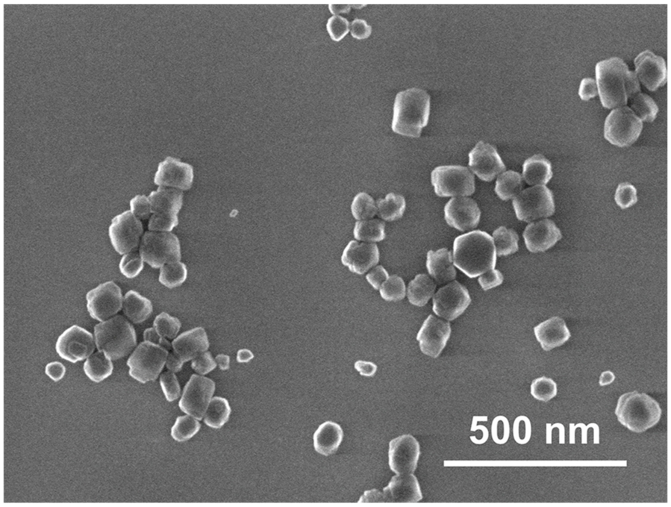

Figure S6. SEM image of silicalite-1 seed crystals.
